# Satellite‐Derived NDVI Predicts Forage Availability in a Wild Ungulate System: Ground‐Truthing Using Field‐Collected Vegetation Biomass

**DOI:** 10.1002/ece3.73258

**Published:** 2026-03-12

**Authors:** Shane Butt, Kirsty Macphie, Richard S. Turner, Sean J. Morris, Alison Morris, Robin Pakeman, Loeske E. B. Kruuk, J. M. Pemberton, H. Froy

**Affiliations:** ^1^ School of Biological Sciences Institute of Ecology and Evolution, University of Edinburgh Edinburgh Scotland; ^2^ James Hutton Institute Aberdeen UK

**Keywords:** biomass, EVI, grazing, Landsat, long‐term population study, MODIS, phenology, red deer *Cervus elaphus*, satellite, vegetation

## Abstract

Satellite‐derived vegetation indices provide a powerful means to quantify habitat variation in long‐term ecological studies, but their reliability as proxies for forage availability in wild herbivore populations remains underexplored. We used three decades of Landsat satellite imagery (1991–2023) to generate a 30 m resolution dataset of a proxy for annual vegetation greenness – the Normalised Difference Vegetation Index (NDVI) – for the Isle of Rum, Scotland, home to a long‐term study of wild red deer ( *Cervus elaphus* ). We ground‐truthed the NDVI data against live vegetation biomass data collected from calcareous grassland, which is preferred by the deer, and compared it with a coarser‐resolution (500 m) MODIS Enhanced Vegetation Index (EVI) metric. Landsat NDVI was positively correlated with both live biomass and EVI, supporting its ecological relevance as a measure of forage availability. All three metrics have increased over the last three decades, indicating a long‐term greening trend, with the higher resolution Landsat dataset revealing variation in the rate of change among vegetation groups, including grassland habitats preferred by deer. These findings suggest an increase in forage availability over time, which may have important consequences for the red deer on Rum. Our approach provides a transferable framework for integrating satellite data with individual‐based field studies, demonstrating how remote sensing can enhance ecological inference in long‐term wildlife research.

## Graphical abstract

This study uses three decades of field‐collected vegetation biomass data to ground‐truth satellite‐derived Normalised Difference Vegetation Index (NDVI) data from Landsat imagery as a proxy for resource availability for wild red deer on the Isle of Rum, Scotland. We show that positive associations between live biomass, NDVI, and coarser‐resolution MODIS Enhanced Vegetation Index (EVI) affirm the utility of these data. All metrics show greening through time, and the 30 m resolution Landsat data further reveal distinct greening trends among vegetation groups, offering detailed spatial insights into habitat changes that may have important consequences for red deer on Rum.

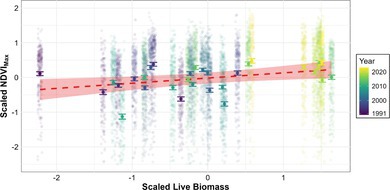

## 1 Introduction

Satellite remote sensing, particularly the use of vegetation indices such as the Normalised Difference Vegetation Index (NDVI), has become an increasingly valuable tool to assess environmental variation in ecological studies of animal populations (Kerr and Ostrovsky 2003; Pettorelli et al. 2005; Cole et al. 2015). NDVI, the most widely used vegetation index in animal ecology studies (Manson et al. 2015; Bahrami et al. 2022), measures greenness and serves as a proxy for vegetation health and coverage (Huete et al. 2002; Pettorelli et al. 2011). It has been linked to variation in foraging conditions, phenology and demography in herbivores (Pettorelli et al. 2006; Hamel et al. 2009; Hurley et al. 2014; Fauchald et al., 2017). However, its reliability as a measure of food availability remains uncertain (Johnson et al. 2018), as NDVI does not distinguish between preferred and unpreferred vegetation, nor does it convey direct information about vegetation quality or quantity. The utility of NDVI as an ecological indicator can also vary depending on habitat type, vegetation structure, and local environmental conditions, making interpretation more complex (Pettorelli et al. 2005; Piedallu et al. 2019). While many studies rely solely on satellite‐derived indices (Rasmussen et al. 2006; Wittemyer et al. 2007; Wiegand et al. 2008; Duffy and Pettorelli 2012; Hurley et al. 2014; Creech et al. 2016; Johnson et al. 2018), few have ground‐truthed these data with field‐based vegetation measures, limiting their ecological interpretability (though see Borowik et al. 2013; Hamel et al. 2009. Who compare NDVI against faecal crude protein, an indicator of vegetation quality). Furthermore, ecologists must be aware of the potential hazards that need to be overcome to utilise remote sensing data from different satellites with confidence (reviewed in Pettorelli et al. 2011).

Many ecological and evolutionary processes unfold over long timescales, making long‐term datasets essential for detecting meaningful trends (Perrins 1965; Grant and Grant 2002; Pucek et al., 2004; Clutton‐Brock and Pemberton 2004; Clutton‐Brock and Sheldon 2010; Ripple and Beschta 2012). The detailed understanding developed over decades of observation lends long‐term population studies significant rigour, making them valuable resources to test fundamental scientific hypotheses in the wild (Reinke et al. 2019). The Isle of Rum red deer ( *Cervus elaphus* ) study is a prime example: ongoing since 1971, it has pioneered research on a range of questions across ecology and evolution in wild mammals (Pemberton et al. 2022). Since 1987, vegetation samples from the calcareous grasslands selectively grazed by deer have been collected alongside long‐term phenotypic, genetic and life‐history data. This offers a rare opportunity to ground‐truth remote sensing vegetation index data and assess NDVI as a proxy for food availability in a wild herbivore system.

Long‐term population studies are particularly powerful for linking environmental variation to ecological and evolutionary processes as climate change drives shifts in vegetation and habitat condition over years and decades (Pacifici et al. 2015; Parmesan 2006; Garant 2020). While individual‐based data on the focal species are often rich, comparable environmental data are typically limited in spatial resolution or ecological relevance (e.g., based only on local weather stations). This limits our understanding of the spatial and temporal dimensions of environmental change and its effects on natural populations. Because vegetation mediates many climate impacts – through changes in plant growth, quality, quantity and seasonality (Thornton et al. 2014) – capturing both the timing and spatial heterogeneity of vegetation change is crucial for studying herbivores, who depend directly on forage availability. By enabling consistent, spatially explicit monitoring of vegetation over time (Pettorelli et al. 2005; Hamel et al. 2009; Santin‐Janin et al. 2009), satellite derived indices such as NDVI offer a valuable complement to ground‐collected data in long‐term population studies.

Previous studies on the Rum red deer have revealed temporal and spatial variation in multiple traits including lifetime breeding success (Rose et al. 1998), parturition dates (Bonnet et al. 2019), vital rates (Coulson et al. 2004) and parasite load (Albery et al. 2018), but the role of vegetation in these patterns remains unclear. To enable future analyses linking spatiotemporal variation in vegetation to red deer performance, we assembled a high spatial resolution NDVI dataset from Landsat remote sensing data (obtained from the U.S. Geological Survey) spanning 1991–2023. To ground‐truth the temporal variation in NDVI in the Landsat dataset, we used monthly measures of above‐ground live vegetation biomass collected from calcareous grassland as part of the long‐term study. We also drew on a pre‐processed product from the Moderate Resolution Imaging Spectroradiometer (MODIS) satellite, measuring the Enhanced Vegetation Index (EVI) at a 500 m spatial resolution from 2000–2022. This provides a well‐validated, internally consistent, single‐sensor reference time series with daily image capture (Gao et al. 2003; Jarchow et al. 2018) to act as an independent check on our Landsat data processing pipeline and the inferred long‐term trends.

The objectives of this study were therefore threefold: (1) to assemble a long‐term, high‐resolution NDVI dataset for the Isle of Rum from Landsat imagery; (2) to evaluate the consistency, robustness and ecological relevance of this dataset by ground‐truthing against long‐term field measurements of vegetation biomass from selectively grazed grassland, and by comparing with a single‐sensor, pre‐processed MODIS EVI product; and (3) using these data, to investigate temporal trends in vegetation on Rum and provide insights into potential ecological changes over time and across the landscape.

## 2 Methods

### 2.1 Study Area

**FIGURE 1:**
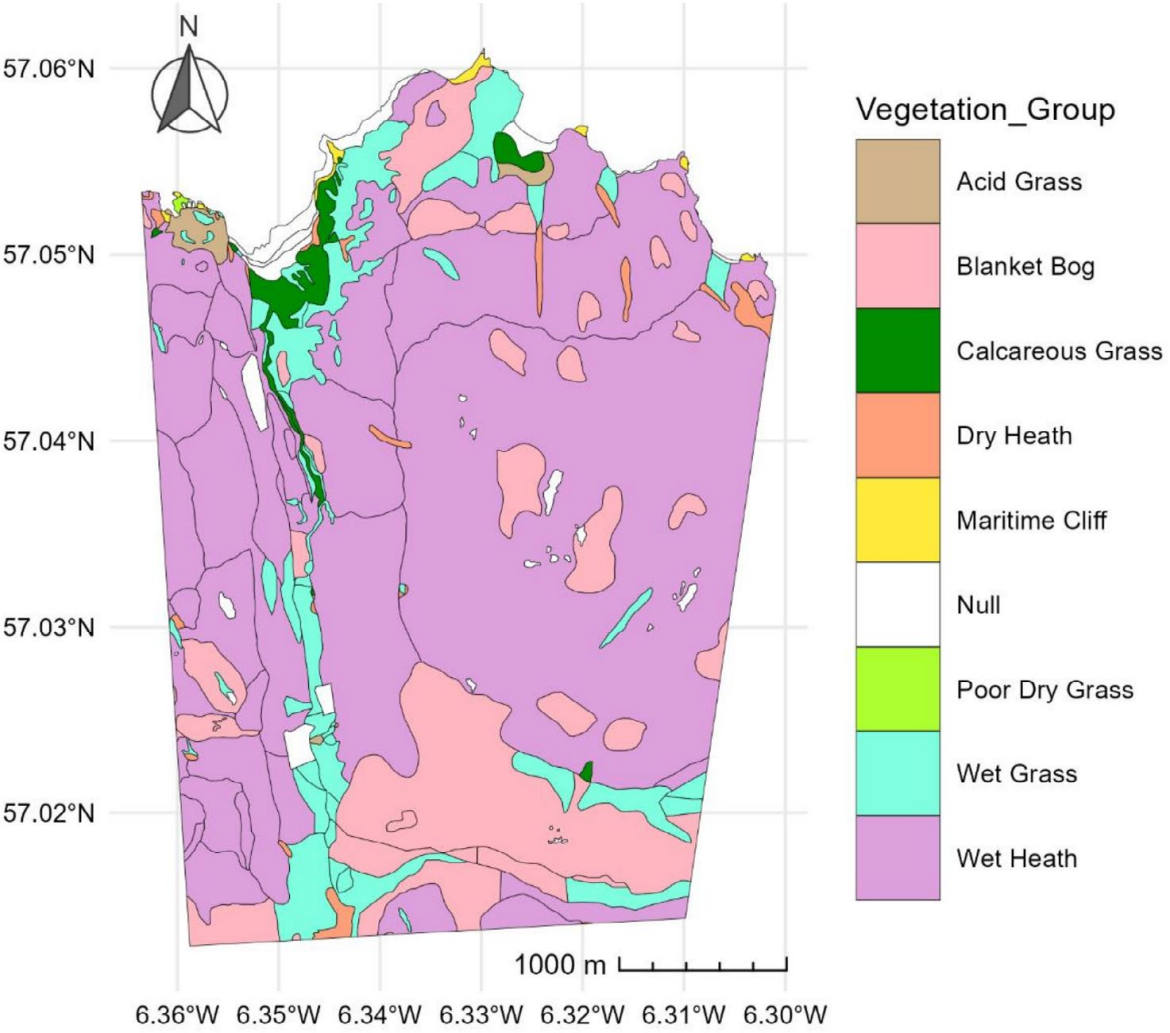
Vegetation map of the Isle of Rum North Block study area, showing the simplified vegetation groups referred to in the text. Compiled using data from the NatureScot Spatial Data Hub.

Rum (57° N, 6°20′W) has a wet, mild oceanic climate. The moorland vegetation is dominated by blanket bog and wet and dry heath interspersed with grassland on better drained areas. The North Block study area (Figure 1) covers ~12.7km^2^ and is divided into different vegetation groups using a map from the NatureScot Spatial Data Hub (https://opendata.nature.scot/datasets/snh::nvc‐habitat‐polygons/explore?location=57.683190%2C‐4.979327%2C6.86), originally commissioned in 1975 and published in 2023. We operate under the assumption that there has been little shift in vegetation group boundaries since 1975. The polygons were mapped to the subgroup level using the National Vegetation Classifications (55 subgroups). To reduce the complexity of the classification we aggregated these subgroups into eight main vegetation groups: acid grass, blanket bog, calcareous grass, dry heath, maritime cliff, poor dry grass, wet grass and wet heath (Figure 1; Table S1; Figure S1). Small portions of the study area remain unclassified because they are tree plots or not vegetated. The predominant vegetation groups in the study area are wet heath and blanket bog, along with smaller patches of dry (and herb‐rich) heaths, acid grassland, wet grass and calcareous grassland (Moore et al. 2015). The calcareous, wet and acid grass areas along the northern coastline and running down the main north–south valley in the study area are preferred by the deer for grazing (Gordon 1989).

### 2.2 Long‐Term Live Vegetation Biomass Measures on Calcareous Grassland

As part of a vegetation monitoring programme focused on the vegetation group most preferred by the deer, calcareous grassland, we have measured the live biomass of grass and herbs each month from March to November inclusive since 1987. At each of six locations – four in the calcareous grass section approximately (57.05° N, 6.35° W) on Figure 1 and two in the calcareous grass section approximately (57.055° N, 6.325° W) – an area of homogeneous vegetation cover approximately 25 × 25 m was chosen. At each location, in each month, ten 10 x 10 cm quadrats located within about 5 m of each other were placed on the ground and the above‐ground vegetation picked down to soil level. The samples from the 10 quadrats at each location were pooled, thoroughly mixed and then approximately 20% was withdrawn and sorted into *live grass & herbs*, *dead grass & herbs*, *moss* and *heather* and then oven‐dried alongside the remaining 80% unsorted, and finally weighed. The proportion of live grass & herbs in the sorted component was used to estimate the weight of live grass and herbs in grams per m^2^ for each site in each month and is hereafter referred to as ‘live biomass’. The protocol ensured that quadrats were not placed on the same spots close in time.

The temporal pattern in live biomass within each year (Figure S2) shows that the annual peak occurred in June or July, so the annual mean live biomass across all six locations in June and July was used as a field‐based estimate of peak‐season above‐ground vegetation biomass. This metric is temporally and conceptually comparable to the NDVI_Max_ (peak vegetation greenness) estimates produced from the Landsat data (see below).

### 2.3 Normalised Difference Vegetation Index and Enhanced Vegetation Index

NDVI is calculated from surface reflectance values in the near‐infrared (NIR) and red spectral regions of satellite images (Carlson & Ripley, 1997; Huete et al. 2002; Pettorelli 2013; Huang et al., 2021). The formula for NDVI is.

(1) $$\text{NDVI}=\frac{\left(\mathrm{NIR}-\mathrm{Red}\right)}{\left(\mathrm{NIR}+\mathrm{Red}\right)},$$

resulting in values between −1 and 1. The NDVI signal arises from strong absorption of red radiation by chlorophyll and high reflectance of NIR radiation by vegetation canopies, such that NDVI reflects not only leaf chlorophyll content but also canopy structure, leaf area, and background soil reflectance (Sellers 1985; Huete et al. 2002; Zou and Mõttus 2017). Healthy and active vegetation typically exhibits high NIR reflectance and low red reflectance, producing high NDVI values, whereas low or negative values generally indicate non‐vegetated surfaces such as water, rock, bare soil, or built areas.

EVI was developed to improve sensitivity in high‐biomass regions and to reduce atmospheric and background soil influences that can limit NDVI performance, particularly in dense canopies. Like NDVI, EVI ranges from −1 to 1 and is derived from red and NIR reflectance, but additionally incorporates the blue band to correct for aerosol scattering and includes terms to adjust for background and canopy effects (Huete et al. 2002; Zou and Mõttus 2017). The formula for EVI is.

(2) $$\mathrm{EVI}=G\frac{\left(\mathrm{NIR}-\mathrm{Red}\right)}{\left(\mathrm{NIR}+C_{1}\mathrm{Red}-C_{2}\text{Blue}+L\right)}$$

where *G* is the gain factor; L is the canopy background adjustment to account for nonlinear, differential NIR and red‐light transfer through the canopy; and C_1_ and C_2_ are the coefficients for the aerosol resistance term (Huete et al. 1994, 2002). By accounting explicitly for atmospheric and background effects, EVI retains greater sensitivity than NDVI in high leaf area index and closed‐canopy conditions, making it particularly suitable for monitoring vegetation dynamics in densely vegetated systems. NDVI and EVI have been shown to be strongly correlated in many studies (Gao et al. 2003; Huete et al. 2002; Vermote et al. 2016; Alademomi et al. 2020).

### 2.4 Landsat Data

We used data from Landsat 5, Landsat 7 and Landsat 8 in our study; Landsat 4 and Landsat 9 were available, but Landsat 4 data are sparse and yielded only two clear images, while Landsat 9 was only launched in 2021. Landsat offers the benefit of relatively high spatial resolution (30 × 30 m) but has a relatively low temporal resolution (16 days) for each satellite. However, because multiple Landsat satellites have operated over the study period with staggered overpass schedules, imagery is available more frequently when data from all sensors are combined. This sampling frequency has doubled since 1999, since when two satellites have operated concurrently. Our raw dataset ranged between March 1984 and December 2023, though the availability and frequency of images varied due to changes in satellite missions. Due to Rum's high latitude and the timing of Landsat satellite overpasses, images are only available from March to October each year. From 1984 to 2012, data were primarily collected from Landsat 5, with additional coverage from Landsat 7 after 1999. However, the failure of Landsat 7's scan line corrector in 2003 introduced significant data gaps (~22% per scene; Storey et al. 2005). The launch of Landsat 8 in 2013 improved data availability, with images captured approximately every 8 days when combined with Landsat 7. Due to low data availability, we excluded the years 1984–1990, 2004 and 2012 from our analyses.

We used the *LandsatTS (v1.2.3)* R package (Berner et al. 2023) to download and process Google Earth Engine‐hosted Level‐2 Collection‐2 Tier‐1 Landsat 5 (Thematic Mapper [TM]), Landsat 7 (Enhanced Thematic Mapper Plus [ETM+]), and Landsat 8 (Operational Land Imager and Thermal Infra‐Red Scanner [OLI‐TIRS]) satellite imagery. We bounded the geographical data range using a vegetation categorised shapefile of the Isle of Rum North Block study area. Each image was pre‐processed to categorise each pixel using the automated function mask (*cfmask*) algorithm (Zhu and Woodcock 2012, 2014). Pixels were categorised as either cloud, cloud shadow, snow, water, or valid. In accordance with Berner et al. 2023, surface reflectance measurements with geometric uncertainty above 30 m were excluded, as were measurements where the solar zenith angle was abnormally high (above 60 m). Further, measurements with physically implausible reflectance values (> 1) and extremely low reflectance (< 0.005) were excluded as a quality‐control step to remove sensor artefacts, deep shadow, water and other noise‐prone pixels that can destabilise NDVI calculations. Values greater than 1 are non‐physical and typically arise from residual atmospheric or calibration errors, while very low reflectance values often correspond to non‐vegetated or poorly illuminated surfaces (Huete et al. 2002; Zhu and Woodcock 2012, 2014; Roy et al. 2014; Mutanga et al. 2023). Images for which > 95% of the pixels were invalid for any reason were discarded entirely as a conservative precaution. These pre‐processing filters reduced the size of the dataset by 87%, from 27.5 million datapoints to 3.6 million; almost all (97%) of the reduction was due to filtering out cloud‐covered pixels. Each valid pixel within the study area was assigned a vegetation group based on the vegetation polygon containing its centroid (Figure 1). This assignment was used only as a categorical label for grouping and modelling. Specific vegetation groups that are not inhabited by deer or lacked sufficient representation (poor dry grass, maritime cliff) and pixels assigned to unmapped areas were removed from the analyses. Pixels containing NDVI values < 0.15 were removed, as values below this threshold typically indicate non‐biomass areas such as rocks, beach, concrete or buildings (Eastman et al., 2013).

#### 2.4.1 Cross‐Calibration between Landsat 5, 7 and 8.

We used the *LandsatTS* package to cross calibrate the surface reflectance measurements between the three satellites (Figure S3). The cross‐calibration process is crucial to avoid introducing artificial trends when analysing temporal NDVI data from multiple Landsat sensors (Roy et al. 2016). In summary, the approach followed the workflow described in Berner et al. (2023) and involved using Landsat 7 and Landsat 5/8 data from overlapping years (1999–2013 for Landsat 5 and Landsat 7; 2013–2023 for Landsat 7 and Landsat 8) to identify corresponding surface reflectance measurements at sample sites, and training a random forest model using 75% of the available data to predict Landsat 7 reflectance based on Landsat 5/8 reflectance values. The remaining 25% of data was used to cross‐validate the model. To overcome the lack of sufficient valid NDVI pixel data for model training, we employed the high‐latitude training dataset provided by the *LandsatTS* package (again following Berner et al. 2023). To account for potential seasonal and spatial differences between sensors, the random forest models include the midpoint of each 15‐day period and the spatial coordinates of each sample as covariates. The spatial coordinates act as flexible proxies for regional effects, allowing the model to capture spatially varying sensor biases rather than imposing a single global calibration (see Berner et al. 2023 for full details of the method). Post cross‐calibration, pixels were on average around 5% ‘greener’ in 2023 compared to 1991, whereas without cross‐calibration this was around 20% (Figure S4).

#### 2.4.2 Phenological Spline Fitting

From the cross‐calibrated data we quantified the growing season characteristics using *LandsatTS*. This process involved iteratively fitting cubic splines to pixel measurements pooled over a seven‐year moving window within the growing season. Further details can be found in (Figure S5). From these splines, we computed vegetation growing season summary statistics. We used the annual NDVI_Max_ for each pixel in our analyses: this is the maximum NDVI value from the peak of the fitted splines. The reason for this step is to account for differences in the seasonal timing of available images to generate a metric that is comparable between years.

### 2.5 MODIS Data

We used data from the MODIS satellite via the pre‐processed MODIS Land Cover Dynamics Version 6.2 (MCD12Q2v062) product, which provides annualised metrics (Friedl et al. 2022). Data for the period 2000–2022 were obtained from Google Earth Engine using the R package *MODISTools* (Hufkens 2022). The product is generated from time series of the 2‐band Enhanced Vegetation Index (EVI2), calculated from MODIS BRDF‐adjusted reflectance (corrected for the effects of varying view and illumination angles on surface reflectance). Outliers are removed, and values during dormant periods are filled. A cubic smoothing spline is fitted to the time series, from which annual phenology metrics are extracted. Full details of the method can be found in the product user guide (Friedl et al. 2022). We did not assign vegetation groups to MODIS pixels due to the coarseness of the 500 m spatial resolution, meaning that any one pixel was likely to encompass several vegetation groups.

Vegetation index metrics in the MODIS product include EVI Amplitude (EVI_Amp_), EVI Minimum (EVI_Min_) and EVI Area (EVI_Area_); we used EVI_Amp_ in our analyses as the best comparison to NDVI_Max_. Although an EVI Maximum metric could theoretically be calculated as the sum of EVI_Amp_ and EVI_Min_, the MODIS product constrains EVI_Min_ to a lower bound of 0.15, potentially introducing skew. Annual phenology metrics are calculated at key stages of the growing season, based on the day of year when EVI first or last crosses specific percentage thresholds of its annual amplitude. The day at which EVI reached its maximum was missing from the data, so we used EVI_MaturityDOY_ – the day at which EVI first crossed 90% of EVI_Amp_ – as a proxy for EVI_MaxDOY_ to investigate phenology trends. Further details on the MCD12Q2 metrics can be found in (Figure S7).

### 2.6 Statistical Analyses

We used linear mixed‐effects models (LMMs) to test for associations between, and temporal trends within, vegetation indices from our Landsat, MODIS and picked vegetation datasets. Analyses were conducted using the *lme4* (Bates et al. 2015) and *glmmTMB* (Brooks et al. 2017) packages in *R 4.3.3 (R Core Team* 2024). Plots were made with *ggplot2* (Wickham 2016).

We first compared our Landsat NDVI measures to the picked vegetation data to act as a ground‐truth. As vegetation sampling locations are restricted to the calcareous grasslands, we used NDVI_Max_ estimates restricted to this vegetation group as the response variable. The mean live biomass across June and July (averaged over locations) was used as the predictor variable, with random intercept terms included to account for repeated measures of pixels and years. Both live biomass and NDVI were z‐standardised (mean = 0, standard deviation = 1). This model included 8003 observations of 267 Landsat pixels and 31 years.

We then compared our Landsat NDVI dataset to the pre‐packaged MODIS EVI dataset. To obtain Landsat NDVI_Max_ values on the same 500 m resolution as MODIS, we calculated the mean NDVI_Max_ of pixels within each MODIS pixel. We removed the outer edge pixels to ensure every MODIS pixel was entirely bound within the study area, and therefore compared like for like with its overlapping Landsat pixels. We used Landsat NDVI_Max_ as the predictor in an LMM with MODIS EVI_Amp_ as the response, incorporating random effects for pixel ID and year. We then ran an additional model to understand whether the association between NDVI_Max_ and EVI_Amp_ was driven by variation among pixels or among years, using a within‐pixel centring approach to decompose the spatial and temporal variation in EVI_Amp_ (van de Pol and Wright 2009). We calculated MeanEVI_Amp_ as the average across all years for each pixel, and RelativeEVI_Amp_ as the deviation of each observation from its pixel‐level mean. MeanEVI_Amp_ captures long‐term spatial differences in EVI_Amp_, while RelativeEVI_Amp_ reflects interannual variation at the pixel scale. Both were included as continuous fixed effects in a LMM of NDVI_Max_, along with random intercepts for pixel and year to account for non‐independence of observations. These models included 958 observations across 46 pixels and 22 years.

Finally, we tested for temporal trends in each of our vegetation metrics. The variable *year* was z‐standardised (mean = 0, standard deviation = 1) to facilitate interpretation of model coefficients. For our picked vegetation data, we ran a LMM of mean live biomass in June and July for each location in each year, with year as a fixed covariate and location as a random intercept to account for non‐independence of locations. This model included 222 observations of 6 locations and 37 years. For the MODIS dataset, we modelled EVI_Amp_ including year as a fixed covariate. and random effects for pixel location and year. We fitted an additional model to test for a temporal trend in EVI_MaturityDOY_, which serves as our best proxy for the date of peak greenness, again using an LMM with fixed effects of year and random effects of pixel and year. The MODIS models included 1403 observations across 64 MODIS pixels and 22 years.

To investigate temporal change in the Landsat data, we fitted a series of LMMs of NDVI_Max_ including year (scaled) as a fixed covariate and random intercepts for pixel ID and year to account for spatial and temporal heterogeneity. To explicitly account for spatial autocorrelation, we included a spatial random effect using an exponential covariance structure based on a position variable created by rounding latitude and longitude coordinates to three decimal places (dividing the study area into a grid of 100 × 100 m cells), which was converted into a numerical factor $S\left({\mathrm{pos}}_{i}\right)$ in the model. We extended this baseline by adding vegetation group as a fixed factor with six levels (blanket bog, wet grass, wet heath, acid grass, calcareous grass, dry heath), producing an additive model. This allowed us to test whether NDVI_Max_ differs among vegetation groups on average. Finally, we fitted an interaction model including the interaction between year and vegetation group, to assess whether temporal trends in NDVI_Max_ vary among vegetation groups. The significance of the additive and interactive effects of vegetation group were assessed using likelihood ratio tests. These models included 511,415 observations from 17,035 pixels and 31 years.

## 3 Results

### 3.1 Landsat NDVIMax vs. Live Biomass

**FIGURE 2:**
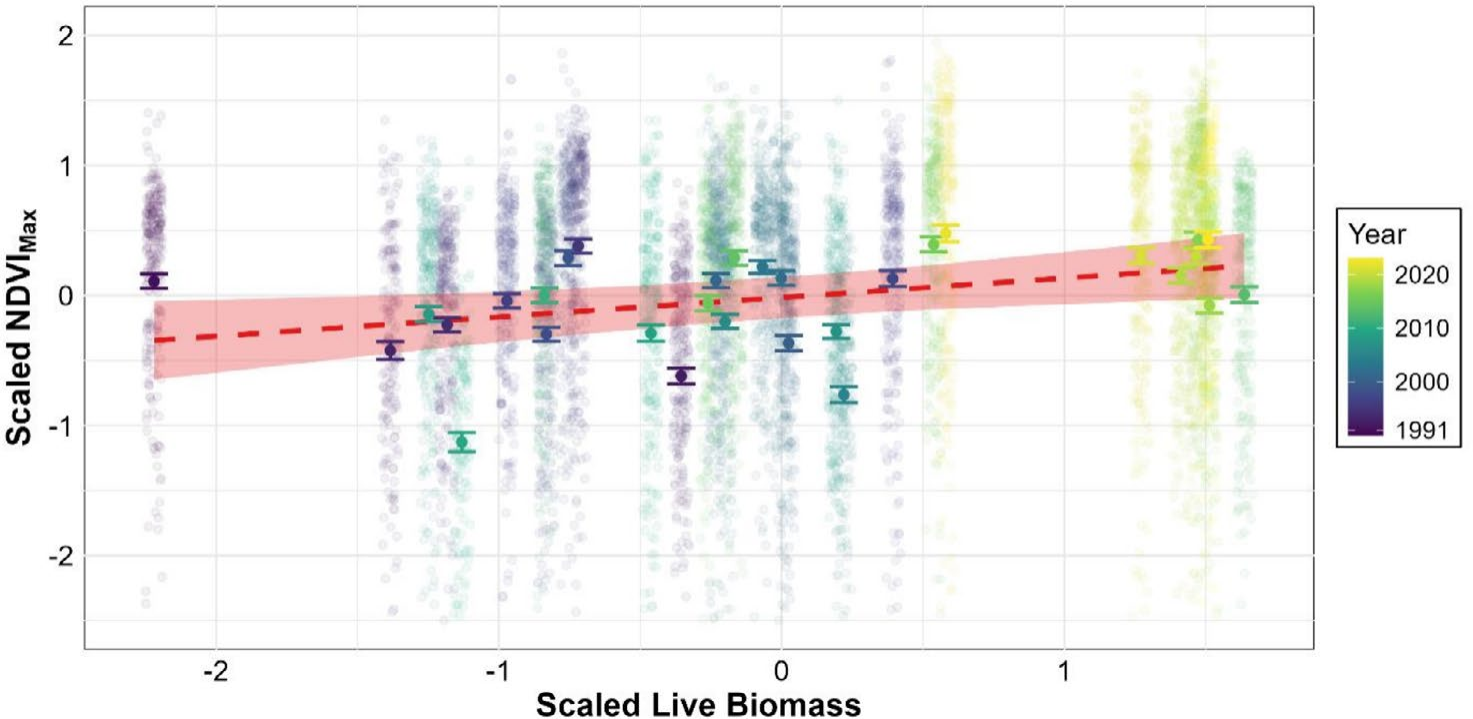
Average annual NDVI_Max_ of calcareous grassland plotted against mean live biomass in June and July on calcareous grassland at six locations within the study area. Both variables have been scaled. Red dashed line indicates model prediction, shaded area indicates 95% confidence interval. Larger points with error bars indicate the mean NDVI_Max_ for a given year, faded points indicate the individual pixel data. Colour indicates year.

There was a significant positive association between NDVI_Max_ and live biomass in June and July: a one standard deviation increase in live biomass is associated with an estimated 0.15 standard deviation increase in NDVI_Max_ (Table 1; Figure 2). While the association is modest, these results indicate that Landsat‐derived NDVI_Max_ captures biologically meaningful variation in above‐ground vegetation biomass on calcareous grassland.

**TABLE 1:** Parameter estimates for a linear mixed model testing the association between live biomass in June and July averaged over locations on calcareous grass and average annual NDVI_Max_ on calcareous grassland, with random intercepts for Landsat pixel and year. Variance components reflect the proportion of total variance attributable to each random effect. Fixed effects significant at the 5% level are highlighted in bold in the *p*‐value column.

Component	Estimate	Std. error	*p*‐value	Variance	% Total Variance
**Fixed effects**					
Intercept	−0.017	0.081	< 0.834	—	—
Live biomass	0.149	0.059	**0.018**	—	—
**Random effects**					
Pixel	—	—	—	0.801	80.0%
Year	—	—	—	0.109	10.9%
Residual	—	—	—	0.091	9.1%

### 3.2 Landsat NDVIMax vs MODIS EVIAmp

There was a significant positive relationship between EVI_Amp_ and NDVI_Max_, indicating that increases in EVI, measured using MODIS, are associated with higher NDVI values measured by Landsat (Table 2).

**TABLE 2:** Parameter estimates for a linear mixed model testing the association between NDVI_Max_ and EVI_Amp_, including random effects of pixel and year. Variance components reflect variance attributable to each random effect. Fixed effects significant at the 5% level are marked in bold in the *p*‐value column.

Component	Estimate	Std. error	*p*‐value	Variance	% Total Variance
**Fixed effects**					
Intercept	0.671	0.011	**< 0.001**	—	—
EVI_Amp_	0.036	0.015	**0.0126**	—	—
**Random effects**					
Pixel	—	—	—	0.00309	79.4%
Year	—	—	—	0.00067	17.2%
Residual	—	—	—	0.00022	3.4%

Decomposing this association into spatial vs. temporal contributions revealed significant positive relationships between NDVI_Max_ and both mean and relative EVI_Amp_. A strong positive association was found between mean EVI_Amp_ and NDVI_Max_, such that pixels with consistently higher EVI values exhibited higher peak NDVI values (Table 3). Additionally, year‐to‐year deviations from a pixel's average EVI_Amp_ were positively associated with NDVI_Max_, though this effect was smaller in magnitude. These findings suggest that both persistent spatial differences in EVI_Amp_ and interannual fluctuations contribute to the positive association in peak vegetation greenness measured by MODIS and Landsat.

**TABLE 3:** Parameter estimates for a linear mixed model testing the association between NDVI_Max_ and EVI_Amp_ using mean and relative EVI_Amp_ to disentangle the spatial and interannual effects, including random effects of year and pixel. Variance components reflect the variance attributable to each random effect. Fixed effects significant at the 5% level are marked in bold in the *p*‐value column.

Component	Estimate	Std. error	p‐value	Variance	% Total Variance
**Fixed effects**					
Intercept	0.379	0.028	**< 0.001**	—	—
Mean EVI_Amp_	0.996	0.090	**< 0.001**	—	—
Relative EVI_Amp_	0.029	0.015	**0.0463**	—	—
**Random effects**					
Pixel	—	—	—	0.00087	53.7%
Year	—	—	—	0.00067	41.5%
Residual	—	—	—	0.00022	4.8%

### 3.3 Temporal Trend in Live Biomass

**FIGURE 3:**
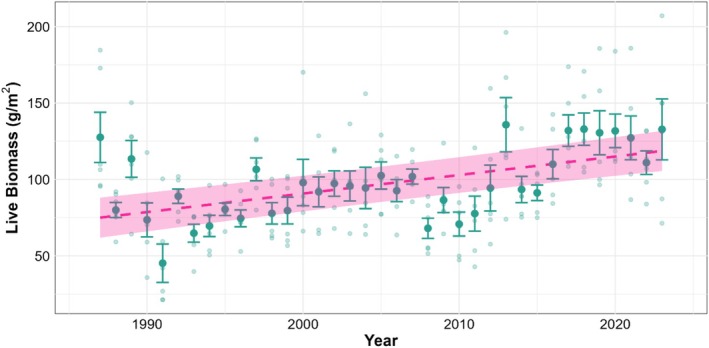
Temporal trend in average mean live biomass in June and July sampled at six locations on calcareous grassland across the study area. Pink dashed line indicates model prediction, with shaded area a 95% confidence interval. Larger points with error bars indicate the average live biomass across the six locations. Smaller, faded points indicate the individual location data.

There was a significant positive effect of year on mean live biomass in June and July (Table 4, Figure 3). This indicates that, on average, the live biomass increased by approximately 1.8 g/m^2^ per year over the study period, after accounting for variability between locations. The random intercept for location accounted for almost 20% of the total variance, reflecting some location‐specific differences in live biomass levels.

**TABLE 4:** Parameter estimates for a linear mixed model of the temporal trend in mean live biomass in June and July. Includes random effect of vegetation sampling location. Fixed effects significant at the 5% level are marked in bold in the *p*‐value column.

Component	Estimate	Std. error	p‐value	Variance	% Total Variance
**Fixed effects**				—	—
Intercept	−23.322	3.478	**< 0.001**	—	—
Year	0.018	0.0019	**< 0.001**	—	—
**Random effects**					
Location (Intercept)	—	—	—	0.0185	19.6%
Residual	—	—	—	0.0761	80.4%

### 3.4 Temporal Trend in MODIS EVIAmp

**FIGURE 4:**
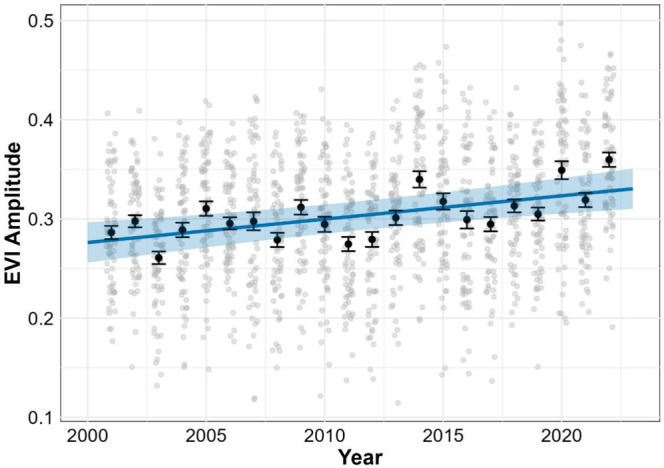
Model estimates of EVI_Amp_ from MODIS data. Grey points show raw pixel‐level EVI_Amp_ values, while black points and error bars represent annual means ±1 SE. The dashed line indicates fitted model predictions and the shaded band indicates the 95% confidence interval.

There was a significant positive trend in EVI_Amp_ over time (Figure 4; Table 5), indicating an overall increase in vegetation greenness across the study area between 2000 and 2022 detected by MODIS. Spatial variability was far greater than inter‐annual variability, reflecting the heterogeneous nature of vegetation across the landscape.

**TABLE 5:** Parameter estimates for the linear mixed model of EVI_Amp_ using MODIS data, including random effects of year and pixel.

Component	Estimate	Std. error	p‐value	Variance	% Total Variance
**Fixed effects**					
Intercept	−4.433	1.260	**0.002**	—	—
Year	0.002	0.001	**0.001**	—	—
**Random effects**					
Pixel	—	—	—	0.00265	65.1%
Year	—	—	—	0.00033	8.1%
Residual	—	—	—	0.00109	26.8%

**FIGURE 5:**
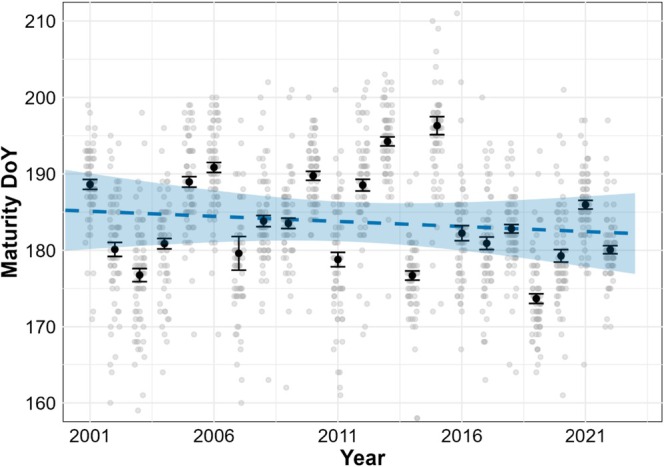
Model estimates of EVI_MaturityDOY_ from MODIS data. Grey points show raw pixel‐level values, while black points and error bars represent annual means ±1 SE. The dashed line indicates fitted model predictions and the shaded band indicates the 95% confidence interval.

There was no significant effect of year on EVI_MaturityDOY_, meaning we found no evidence that the phenology of the growing season peak has changed in the period 2000–2022 (Figure 5; Table 6). Random effects revealed substantial inter‐annual variability compared to spatial variability, indicating that year‐to‐year environmental factors had a stronger influence on maturity than spatial heterogeneity.

**TABLE 6:** Parameter estimates for the linear mixed model predicting vegetation maturity (used as a proxy for the date EVI reached its peak) using MODIS data. EVI_MaturityDOY_ is modelled as a function of year (scaled), with random intercepts for pixel and year. Variance components reflect the variance attributable to each random effect.

Component	Estimate	Std. error	p‐value	Variance	% Total Variance
**Fixed effects**				—	—
Intercept	183.721	1.316	**< 0.001**	—	—
Year (scaled)	−0.842	1.296	0.523	—	—
**Random effects**					
Pixel	—	—	—	3.556	3.8%
Year	—	—	—	36.135	38.5%
Residual	—	—	—	54.274	57.8%

### 3.5 Temporal Trends in Landsat NDVIMax

Model comparison using likelihood ratio tests revealed significant improvements in model fit with increasing complexity. Including vegetation group as a fixed effect significantly improved the model compared to the baseline without vegetation groups (χ^2^ = 160.68, df = 5, *p* < 0.001), indicating that NDVI_Max_ differs among vegetation groups. Adding the interaction between year and vegetation group significantly improved fit again (χ^2^ = 426.66, df = 5, *p* < 0.001), demonstrating that temporal trends in NDVI_Max_ vary across vegetation groups.

**FIGURE 6:**
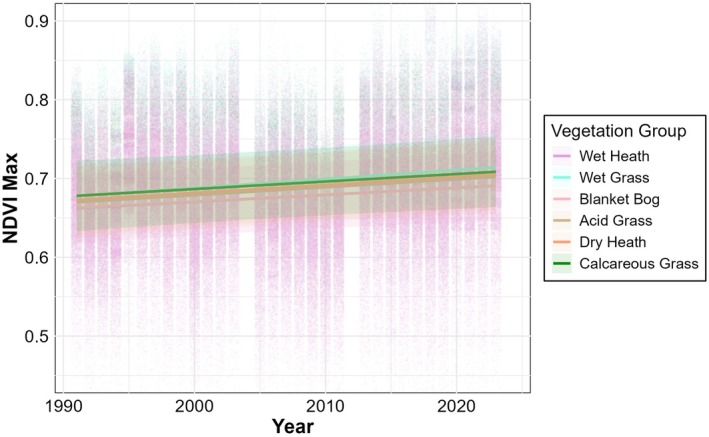
Model estimates of NDVI_Max_ by vegetation group from Landsat data. Individual pixel estimates are coloured by vegetation group. Trendline predictions from the model with vegetation group and year interactions are overlaid. Shaded areas indicate the 95% confidence intervals.

The final interaction model showed a small but significant positive overall effect of year, reflecting a general greening trend across the study period (Figure 6; Table 7). Wet grassland exhibited significantly higher NDVI_Max_ than the reference vegetation group, acid grassland, while other vegetation groups did not differ significantly at the 5% level. Random effect variance partitioning highlights substantial spatial and temporal heterogeneity in vegetation greenness, with the spatial component dominating the overall model variance structure (Table 7). This highlights the importance of spatial structure in explaining NDVI variation. The significant interaction terms revealed contrasting temporal trends among vegetation groups (Figure 6; Figure S6; Table 7). Wet grassland exhibited a significantly stronger increase in NDVI_Max_ over time compared to acid grassland, while blanket bog and wet heath showed significant negative interactions, indicating a slower increase in NDVI_Max_ in these groups which are less preferred by deer. Other interaction terms were not statistically significant. These results suggest that temporal trends in vegetation greenness are not uniform across vegetation groups, with wet grassland in particular greening more rapidly than other groups (this can be seen more clearly in Figure S8).

**TABLE 7:** Parameter estimates for the linear mixed model of NDVI_Max_ using Landsat data. NDVI is modelled as a function of year (scaled) and vegetation group, with an interaction term included. Acid Grass is the reference vegetation group. Significant fixed effects are marked in bold in the *p*‐value column. Variance components reflect the variance attributable to each random effect.

Component	Estimate	Std. error	p‐value	Variance	% Total Variance
**Fixed effects**					
Intercept	0.686	0.022	**< 0.001**	—	—
Year (scaled)	0.0099	0.0043	**0.023**	—	—
Blanket Bog	−0.0091	0.0052	0.083	—	—
Calcareous Grass	0.0075	0.0058	0.197	—	—
Dry Heath	0.0029	0.0059	0.617	—	—
Wet Grass	0.0102	0.0051	**0.047**	—	—
Wet Heath	−0.0093	0.0051	0.067	—	—
Year × Blanket Bog	−0.0013	0.0004	**0.003**	—	—
Year × Calcareous Grass	−0.0005	0.0005	0.310	—	—
Year × Dry Heath	0.0000	0.0005	0.974	—	—
Year × Wet Grass	0.0013	0.0004	**0.003**	—	—
Year × Wet Heath	−0.0011	0.0004	**0.011**	—	—
**Random effects**					
Spatial	—	—	—	0.00559	72.0%
Pixel	—	—	—	0.00099	12.8%
Year	—	—	—	0.00057	7.4%
Residual	—	—	—	0.00061	7.8%

## 4 Discussion

To assess the reliability and ecological relevance of satellite‐derived NDVI as a proxy for deer forage availability, we ground‐truthed our Landsat‐derived NDVI measures with long‐term field‐picked live biomass data: we showed that, on calcareous grassland selectively grazed by deer, annual NDVI_Max_ was positively associated with mid‐summer (June–July) live biomass, confirming that the remote‐sensed data correspond meaningfully to on‐the‐ground vegetation change. Although the Landsat dataset underwent extensive pre‐processing – particularly cross‐sensor calibration and phenological spline fitting – we find further reassurance from its significant association with a pre‐processed, single‐sensor MODIS EVI product. This supports the methodological soundness of our Landsat pipeline and its utility for assessing peak vegetation greenness at finer spatial resolution.

Our ground‐truth findings align with the conclusions of Borowik et al. (2013), who emphasised the need for field validation when using NDVI as a proxy for forage availability in eastern Poland. Their study demonstrated a strong positive relationship between NDVI and ground vegetation biomass in open ‘field’ habitats during summer, comparable to our findings in Rum's calcareous grasslands. However, their study only collected biomass data across 2 years, in 2007 and 2008, a relatively limited time frame for understanding longer‐term trends. In contrast, our study incorporates biomass data collected over three decades, providing a more robust and temporally comprehensive ground‐truth of NDVI as a proxy for vegetation biomass; in this respect, our study contributes a valuable case where NDVI‐derived greenness is meaningfully grounded in direct measures of vegetation biomass. This ground‐truthing confirms that remotely sensed indices can serve as ecologically relevant proxies for vegetation greenness in this habitat. However, it is important to note that our ground‐truth exercise is restricted to calcareous grassland, and therefore does not capture potential variation in NDVI–biomass relationships across all vegetation types present on Rum. NDVI–biomass relationships are known to be context‐dependent, varying with vegetation structure, canopy closure and species composition (Huete et al. 2002; Zou et al., 2017), and caution is therefore required when extrapolating these findings beyond open grassland systems. Nonetheless, calcareous grasslands represent a key foraging habitat for red deer on Rum, and the combination of long‐term biomass data with high‐resolution satellite imagery provides a uniquely robust ground‐truth of the use of NDVI in an ecological context.

We provide evidence that annual peak vegetation greenness on the Isle of Rum has increased over the past almost 40 years, as indicated by significant temporal trends in models of Landsat NDVI, MODIS EVI, and live biomass calculated from field‐measured vegetation data. This suggests long‐term shifts in vegetation greening, with potentially important implications for the red deer feeding on it. For example, individual home range NDVI_Max_ is associated with lower gastrointestinal helminth egg counts (Hasik et al., 2025). Our results suggest that resource availability for deer has generally improved over time, but the ecological consequences depend on how different vegetation groups – both those preferred by deer and those they avoid – have responded to these trends. Our analyses revealed small but significant variations in the rate of change in NDVI_Max_ among different vegetation groups. This suggests that while overall greening trends are apparent, the extent and rate of vegetation change are not uniform across the landscape; this is consistent with a recent study focused on the arctic tundra biome (Berner et al. 2020) and wider global greening trends (Cortés et al. 2021; Correa‐Díaz et al. 2021). Among deer‐preferred habitats, acid grassland shows a clear increase in NDVI_Max_ over time (see Table 7: acid grassland is the reference group) and only wet grassland has a significantly steeper rate of greening than acid grassland. Calcareous grassland, though consistently among the greenest habitats, showed no significant difference in slope to acid grass; this may reflect strong grazing pressure limiting vegetation growth in this area (Zhao et al. 2022), as it is most preferred by the deer. However, it is not necessarily the case that grazing is a primary factor determining NDVI (Garcia et al. 2025). Blanket bog and wet heath are greening more slowly, although still showing positive overall trends. Greening in unpreferred habitats may provide clearer signals of broader environmental change, indicating that system‐wide drivers (i.e., higher ambient temperatures) are likely enhancing vegetation productivity across the landscape. These patterns broadly align with recent findings for Soay sheep ( *Ovis aries* ) on St. Kilda (Pakeman et al. 2024), where preferred forage habitats showed more pronounced greening than less‐preferred types.

Our findings provide important ecological context for changes observed in the deer population. For instance, Moyes et al. (2011) and Bonnet et al. (2019) detected a two‐week advance in red deer parturition dates over recent decades. Our evidence of long‐term increases in vegetation greenness – particularly in habitats favoured by deer – offers a plausible ecological mechanism that could support such phenological shifts, by way of more food leading to better condition and therefore potentially earlier oestrus. A significant advantage of the Landsat dataset is the ability to analyse vegetation dynamics at a much finer spatial resolution than was previously possible in our study system. By generating a 30 m resolution dataset of the study area, we can capture spatial variation in vegetation, providing a more detailed understanding of habitat quality and its potential effects on red deer ecology. Future studies can capitalise on this relatively high spatial resolution by linking it with individual‐level data on deer locations, enabling us to conduct individual‐level analyses that explore how spatiotemporal variation in vegetation influences life‐history traits and population dynamics. Although beyond the immediate scope of this study, we expect the vegetation dataset we have generated to be valuable for a wide range of future analyses, including investigations into movement patterns, foraging decisions, parasite dynamics (Hasik et al., 2025), habitat selection, and climate‐driven ecological changes on Rum.

Although we detected clear trends in peak greenness, we found no reliable evidence of shifts in vegetation phenology over time. This lack of signal may reflect data limitations rather than a true absence of phenological change, especially given well‐documented trends towards earlier spring phenology (Parmesan 2007). On Rum, the presence of data gaps due to poor atmospheric conditions, particularly cloud cover, heavily impacted the availability of high‐quality Landsat images. Additionally, the scan line correction fault on Landsat 7 from 2003 (Storey et al. 2005), corrected in 2012, resulted in missing data, requiring the use of ‘ghost observations’ borrowed from neighbouring years. While this approach helps fill in gaps, it introduces temporal autocorrelation, limiting the reliability of among‐year analyses and potentially affecting short‐term trends. We opted not to model phenology using Landsat data due to concerns over robustness driven by data gaps and sparse temporal coverage, which resulted in unreliable estimates of the day of the year when NDVI_Max_ was reached. Frequent cloud cover also likely reduced the quality and quantity of observations used to construct the annualised MODIS product, limiting our ability to detect trends with confidence – though this is somewhat offset by the data being collected daily, which was a key factor in our decision to also compare our Landsat dataset with a pre‐processed MODIS dataset. There was high interannual variation in EVI_MaturityDOY_, which may indicate a degree of noise or instability in these data. This suggests that the phenological spline fitting process used to derive these metrics could be sensitive to input variation and may introduce artefactual patterns – a concern we also encountered when fitting splines to our Landsat data. We were unable to formally assess the sensitivity of these MODIS‐derived phenology metrics to data density or quality, as the raw input data were not available. The dearth of cloud‐free images, combined with the need to interpolate missing data, reduces the accuracy of detecting precise seasonal shifts in vegetation growth. Annual average vegetation indices are relatively coarse metrics which are perhaps more easily estimable with sparse data than phenology metrics such as the precise timing of green‐up, which could be heavily influenced by a single datapoint. This highlights the trade‐offs inherent in using remote sensing data for ecological studies: while the long‐term trends are robust, short‐term or highly seasonal patterns may be less reliable, and the data are not appropriate for these types of analyses. A potential solution for future analyses is to incorporate imagery from the Sentinel‐2 satellites, which offers a higher spatial resolution of 10 × 10 m and captures images every 5 days, increasing the likelihood of obtaining cloud‐free observations. The improved temporal frequency and finer spatial scale would enhance the accuracy of vegetation monitoring. However, Sentinel‐2 data are only available from 2016, meaning it currently lacks the long‐term historical coverage provided by Landsat, limiting its use for assessing vegetation trends over multiple decades.

Our study further highlights the crucial role of cross‐calibration in maintaining temporal consistency across satellite datasets (Berner et al. 2020). This process is not a minor technical step – it is foundational to ensuring that observed trends reflect ecological reality rather than artefacts of differing sensor sensitivities or spectral responses. We demonstrated that across Landsat sensors, substantial differences in NDVI measures can arise without proper calibration. This introduces the risk of misinterpreting shifts in vegetation dynamics if methodological discrepancies are mistaken for real‐world change. As more high‐resolution Earth observation data become available from platforms like Sentinel‐2 and upcoming missions, ensuring compatibility across sensors will be increasingly vital for robust, multi‐decadal ecological analyses. Our findings reinforce the notion that methodological rigour in calibration is not just good practice – it is a precondition for credible inference about long‐term environmental change.

## 5 Conclusions

Our study demonstrates that peak vegetation greenness on Rum has increased over the past three decades, with this trend evident across two satellite‐based systems and ground‐truthed through long‐term vegetation data collected in the field. These findings suggest an overall increase in annual peak vegetation greenness, with potential consequences, particularly in improving foraging conditions for red deer. While not all vegetation groups have changed at the same rate, habitats favoured by deer – such as acid and wet grasslands – have seen some of the most marked greening, offering a plausible ecological mechanism for changes observed in the population, such as earlier calving (Bonnet et al. 2019).

By ground‐truthing remotely sensed NDVI data with field‐based measures of vegetation on a temperate grassland, our study provides a rare ground‐truth for a satellite‐derived vegetation metric often used in wild population studies (Wittemeyer et al., 2006; Wiegand et al. 2008; Duffy and Pettorelli 2012; Hurley et al. 2014; Creech et al. 2016; Johnson et al. 2018). This strengthens confidence in the ecological relevance of long‐term remote sensing data and highlights the potential for integrating satellite observations with detailed, individual‐based data in wild animal populations. Our work also underscores the methodological challenges of using satellite remote sensing in cloud‐prone, heterogeneous landscapes, particularly for detecting phenological trends.

A key strength of our study lies in the spatial resolution achieved through Landsat data, allowing vegetation change to be assessed at the 30 m pixel scale across the study area. This resolution enables us to quantify forage availability in individual deer habitats through existing long‐term census and location data, enabling individual level analyses of the effects of spatio‐temporal variation in forage. The resulting dataset represents a valuable resource for future research on red deer ecology, with potential applications across demography, life‐history traits, parasite interactions, and responses to climate‐driven environmental change.

Beyond its immediate application to the Rum system, our study offers a practical blueprint for integrating satellite‐derived vegetation indices with field‐based validation in long‐term ecological animal research. By demonstrating how to overcome challenges related to sensor calibration, cloud cover, and phenology extraction, we provide a replicable framework for using remote sensing to monitor ecological change with biological credibility. As high‐resolution Earth observation platforms like Sentinel‐2 continue to improve data availability, such approaches will be increasingly important for understanding how ecosystems respond to environmental change over time and space.

Ultimately, our findings illustrate both the power and the limitations of remote sensing in long‐term ecological research. With careful calibration and interpretation, satellite data can reveal meaningful environmental change across decades, as it has on Rum, enhancing our understanding of how populations respond to climatic and biotic pressures over time.

## Author Contributions

**Shane Butt:** data curation (lead), formal analysis (lead), methodology (lead), validation (lead), visualization (lead), writing – original draft (lead). **Kirsty Macphie:** formal analysis (supporting), visualization (supporting), writing – review and editing (equal). **Richard S. Turner:** data curation (supporting), investigation (supporting), methodology (supporting), validation (supporting), writing – review and editing (equal). **Sean J. Morris:** data curation (equal), investigation (equal), methodology (supporting), validation (supporting). **Alison Morris:** data curation (equal), investigation (equal), methodology (supporting), validation (supporting). **Robin Pakeman:** methodology (supporting), resources (supporting), writing – review and editing (supporting). **Loeske E. B. Kruuk:** conceptualization (equal), funding acquisition (lead), project administration (supporting), supervision (supporting), writing – review and editing (supporting). **J. M. Pemberton:** conceptualization (supporting), funding acquisition (equal), project administration (equal), supervision (supporting), writing – review and editing (supporting). **H. Froy:** conceptualization (supporting), supervision (lead), writing – review and editing (lead).

## Funding

This work was supported by the European Research Council, 101020503. Natural Environment Research Council, NE/X000346/1.

## Conflicts of Interest

The authors declare no conflicts of interest.

## Supporting information


**AppendixS1:** ece373258‐sup‐0001‐AppendixS1.docx.
**Figure S1:** Vegetation map of the study area by National Vegetation Classifications obtained from the NatureScot Spatial Data Hub.
**Figure S2:** Average dry weight of live biomass for each month in each year plotted across months, colour depicts year. The data are shown per 10 cm × 10 cm quadrat, which was then multiplied by 100 to give a value per m^2^ used in analysis.
**Figure S3:** Correlations between (a) Landsat 5 and 7, and (b) Landsat 7 and 8, pre (left‐hand panels) and post (right‐hand panels) cross‐calibration using a random forest model. Orange lines depict one‐to‐one correlations.
**Figure S4:** Histograms of relative NDVI_Max_ change per pixel between 1991 and 2023 – the first and last years of data collection. The orange histogram shows NDVI values with cross‐calibration applied; the green histogram shows values without cross‐calibration.
**Figure S5:** Seasonal NDVI progression for each year from 1985 to 2023 for nine randomly selected pixels in the Isle of Rum study area. Points and their corresponding fitted phenological curves (cubic splines) are colour coded by year. Plot produced using *LandsatTS* package prior to removal of pixels from splines which didn't reach a peak.
**Figure S6:** Annual NDVI_MaxDOY_ model predictions compared against the number of images available for each year. Colour depicts year; red dots overlaid indicate the estimated NDVI_MaxDOY_ for that year. A significant negative relationship was detected: the more images available, the lower the estimate of NDVI_MaxDOY_.
**Figure S7:** Diagram of the vegetation index and phenological metrics outputted by the MODIS MCD12Q2 product, taken from the product user guide (Friedl et al. 2022).
**Figure S8:** Model estimates of NDVI_Max_ by vegetation group from Landsat data. Individual pixel estimates are coloured by vegetation group. Trendline predictions from the model with vegetation group and year interactions are overlaid.
**Table S1:** National Vegetation Classifications for the Isle of Rum based on a map obtained from the NatureScot Spatial Data Hub, including area coverage.

## Data Availability

The data and code that support the findings of this study are openly available using the following DOI: https://doi.org/10.5061/dryad.bzkh189rd.
