# Supplementary material for: Satellite‐Derived NDVI Predicts Forage Availability in a Wild Ungulate System: Ground‐Truthing Using Field‐Collected Vegetation Biomass
Source: Ecol Evol. 2026 Mar 12;16(3):e73258. doi: 10.1002/ece3.73258 (PMC13093347; doi:10.1002/ece3.73258)
Supplement: Supplementary file 1 — AppendixS1: ece373258‐sup‐0001‐AppendixS1.docx. Figure S1: Vegetation map of the study area by National Vegetation Classifications obtained from the NatureScot Spatial Data Hub. Figure S2: Average dry weight of live biomass for each month in each year plotted across months, colour depicts year. The data are shown per 10 cm × 10 cm quadrat, which was then multiplied by 100 to give a value per m2 used in analysis. Figure S3: Correlations between (a) Landsat 5 and 7, and (b) Landsat 7 and 8, pre (left‐hand panels) and post (right‐hand panels) cross‐calibration using a random forest model. Orange lines depict one‐to‐one correlations. Figure S4: Histograms of relative NDVIMax change per pixel between 1991 and 2023 – the first and last years of data collection. The orange histogram shows NDVI values with cross‐calibration applied; the green histogram shows values without cross‐calibration. Figure S5: Seasonal NDVI progression for each year from 1985 to 2023 for nine randomly selected pixels in the Isle of Rum study area. Points and their corresponding fitted phenological curves (cubic splines) are colour coded by year. Plot produced using LandsatTS package prior to removal of pixels from splines which didn't reach a peak. Figure S6: Annual NDVIMaxDOY model predictions compared against the number of images available for each year. Colour depicts year; red dots overlaid indicate the estimated NDVIMaxDOY for that year. A significant negative relationship was detected: the more images available, the lower the estimate of NDVIMaxDOY. Figure S7: Diagram of the vegetation index and phenological metrics outputted by the MODIS MCD12Q2 product, taken from the product user guide (Friedl et al. 2022). Figure S8: Model estimates of NDVIMax by vegetation group from Landsat data. Individual pixel estimates are coloured by vegetation group. Trendline predictions from the model with vegetation group and year interactions are overlaid. Table S1: National Vegetation Classifications for the [file ECE3-16-e73258-s001.docx]

Supplementary Information

**Vegetation Groupings**

We used a base map of the vegetation available from the NatureScot Spatial Data Hub with polygons mapped using the National Vegetation Classifications (NVCs) identified at the subcommunity level. There were 55 subcommunities which map into 32 NVC subgroups; these are further aggregated into 11 vegetation groups, 9 of which are found inside the North Block study area. Table 1 lists the communities found on Rum and their area, both on the island as a whole and within the North Block study area.

| **Vegetation Group** | **NVC** | **NVC Description** | **Found in North Block?** | **Total Area (m^2^)** | **Area in North Block (m^2^)** |
| --- | --- | --- | --- | --- | --- |
| Acid Grass | U4 | Festuca ovina-Agrostis capillaris-Galium saxatile grassland | 1 | 3,057,122 | 103,890 |
| Blanket Bog | M17 | Scirpus cespitosus-Eriophorum vaginatum blanket mire | 1 | 13,744,544 | 4,742,267 |
| Blanket Bog | M1 | Sphagnum auriculatum bog pool community | 1 | 4,412 | 1,323 |
| Calcareous Grass | CG10 | Festuca ovina-Agrostis capillaris-Thymus praecox grassland | 1 | 2,535,207 | 237,867 |
| Dry Heath | H10 | Calluna vulgaris-Erica cinerea heath | 1 | 13,006,348 | 331,680 |
| Dry Heath | H21 | Calluna vulgaris-Vaccinium myrtillus-Sphagnum capillifolium heath | 1 | 109,263 | 10,032 |
| Dry Heath | H11 | Calluna vulgaris-Carex arenaria heath | 1 | 7,141 | 7,141 |
| Dry Heath | H7 | Calluna vulgaris-Scilla verna heath | 1 | 5,452 | 1,980 |
| Maritime Cliff | MC10 | Festuca rubra-Plantago spp. maritime grassland | 1 | 204,373 | 49,313 |
| Null | Null | NA - not categorised | 1 | 6,619,550 | 353,601 |
| Null | U20 | Pteridium aquilinum-Galium saxatile community | 1 | 407,073 | 3,431 |
| Poor Dry Grass | U5 | Nardus stricta-Galium saxatile grassland | 1 | 1,705,957 | 6,178 |
| Wet Grass | M25 | Molinia caerulea-Potentilla erecta mire | 1 | 6,172,745 | 2,127,229 |
| Wet Grass | M23 | Juncus effusus/acutiflorus-Galium palustre rush-pasture | 1 | 643,726 | 345,480 |
| Wet Grass | M6 | Carex echinata-Sphagnum recurvum/auriculatum mire | 1 | 53,263 | 12,120 |
| Wet Heath | M15 | Scirpus cespitosus-Erica tetralix wet heath | 1 | 60,331,491 | 15,746,162 |
| Acid Grass | MG6 | Lolium perenne-Cynosurus cristatus grassland | 0 | 38,706 | - |
| Alpine Heath | U10 | Carex bigelowii-Racomitrium lanuginosum moss-heath | 0 | 170,112 | - |
| Alpine Heath | U7 | Nardus stricta-Carex bigelowii grass-heath | 0 | 62,314 | - |
| Blanket Bog | M10 | Carex dioica-Pinguicula vulgaris mire | 0 | 88,710 | - |
| Calcareous Grass | CG11 | Festuca ovina-Agrostis capillaris-Alchemilla alpina grassland | 0 | 124,982 | - |
| Calcareous Grass | CG12 | Festuca ovina-Alchemilla alpina-Silene acaulis dwarf-herb community | 0 | 2,621 | - |
| Dry Heath | H20 | Vaccinium myrtillus-Racomitrium lanuginosum heath | 0 | 1,016,504 | - |
| Dry Heath | H14 | Calluna vulgaris-Racomitrium lanuginosum heath | 0 | 593,093 | - |
| Dry Heath | H18 | Vaccinium myrtillus-Deschampsia flexuosa heath | 0 | 65,030 | - |
| Maritime Cliff | MC8 | Festuca rubra-Armeria maritima maritime grassland | 0 | 15,387 | - |
| Null | U17 | Luzula sylvatica-Geum rivale tall-herb community | 0 | 25,784 | - |
| Poor Dry Grass | U6 | Juncus squarrosus-Festuca ovina grassland | 0 | 155,255 | - |
| Wet Grass | M32 | Philonotis fontana-Saxifraga stellaris spring | 0 | 129 | - |
| Woodland | W11 | Quercus petraea-Betula pubescens-Oxalis acetosella woodland | 0 | 53,163 | - |
| Woodland | W23 | Ulex europaeus-Rubus fruticosus scrub | 0 | 12,312 | - |
| Woodland | W17 | Quercus petraea-Betula pubescens-Dicranum majus woodland | 0 | 9,734 | - |

**Table S1:** National Vegetation Classifications for the Isle of Rum based on a map obtained from the NatureScot Spatial Data Hub, including area coverage.


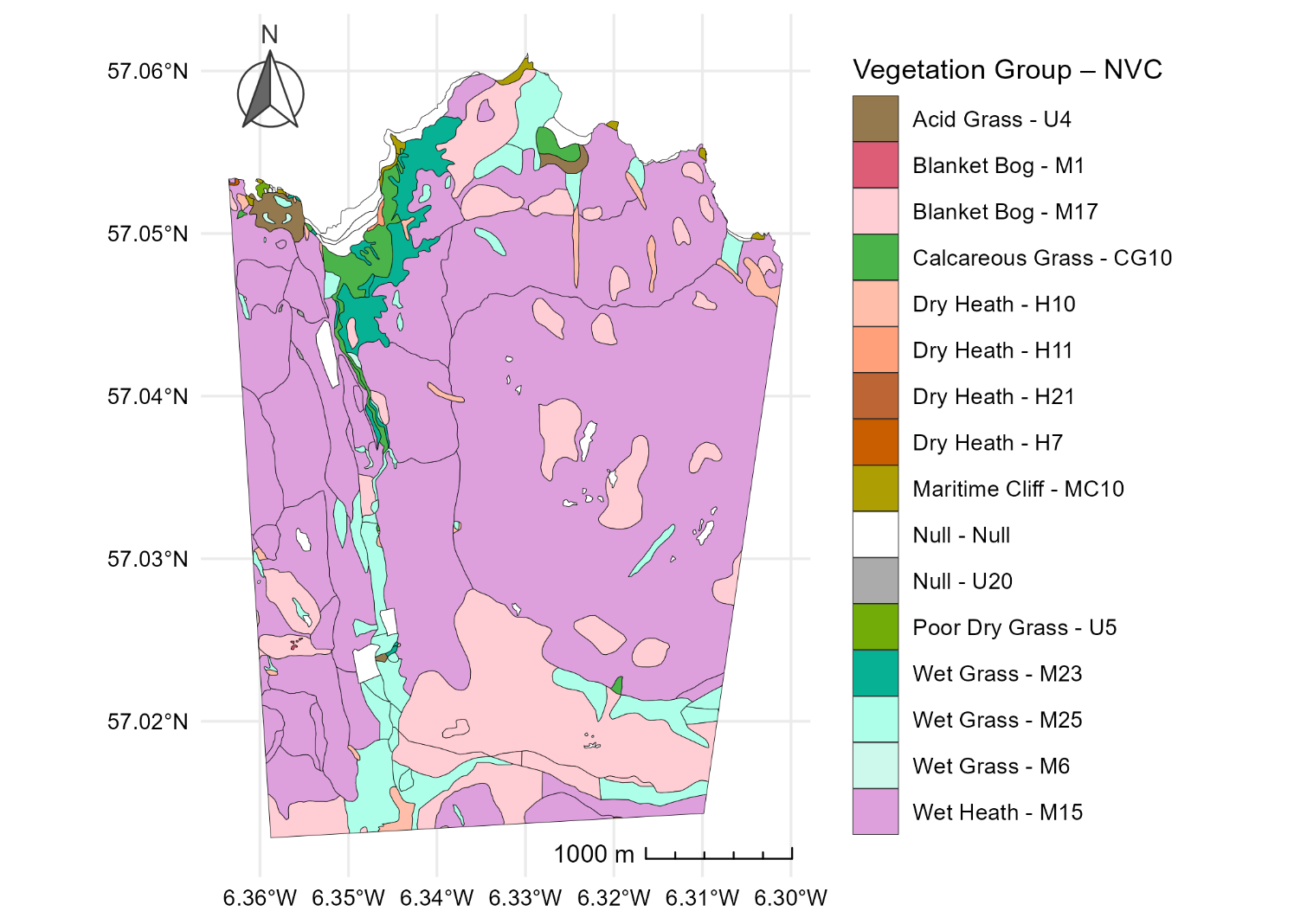


**Figure S1:** Vegetation Map of the study area by National Vegetation Classifications obtained from the NatureScot Spatial Data Hub.

**Long-term live biomass measures on calcareous grassland**

There were no live biomass data collected for September and November 1987, November 1988, August 2003, November 2007 and May 2020. One location wasn’t sampled in May 2007 or June 2023, and on a few occasions only 5 quadrats were sampled. All values were adjusted to calculate live biomass per m^2^

The temporal pattern in live biomass within each location and year (Fig S2) shows the annual peak in live biomass occurred in June or July, so the mean live biomass across June and July for each year was used.

**Figure S2:** Average dry weight of live biomass for each month in each year plotted across months, colour depicts year. The data are shown per 10cm x 10cm quadrat, which was then multiplied by 100 to give a value per m^2^ used in analysis.

**Landsat 5, 7 and 8 Cross Calibration**


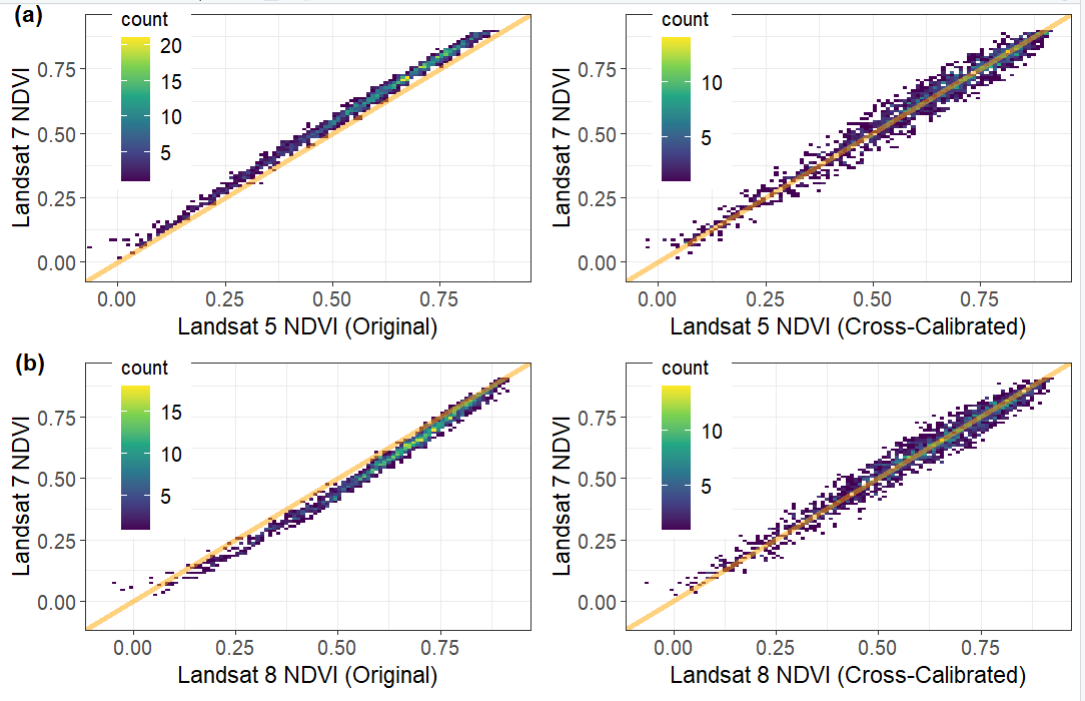


**Figure S3:** Correlations between (a) Landsat 5 and 7, and (b) Landsat 7 and 8, pre (left-hand panels) and post (right-hand panels) cross-calibration using a random forest model. Orange lines depict one-to-one correlations.

**
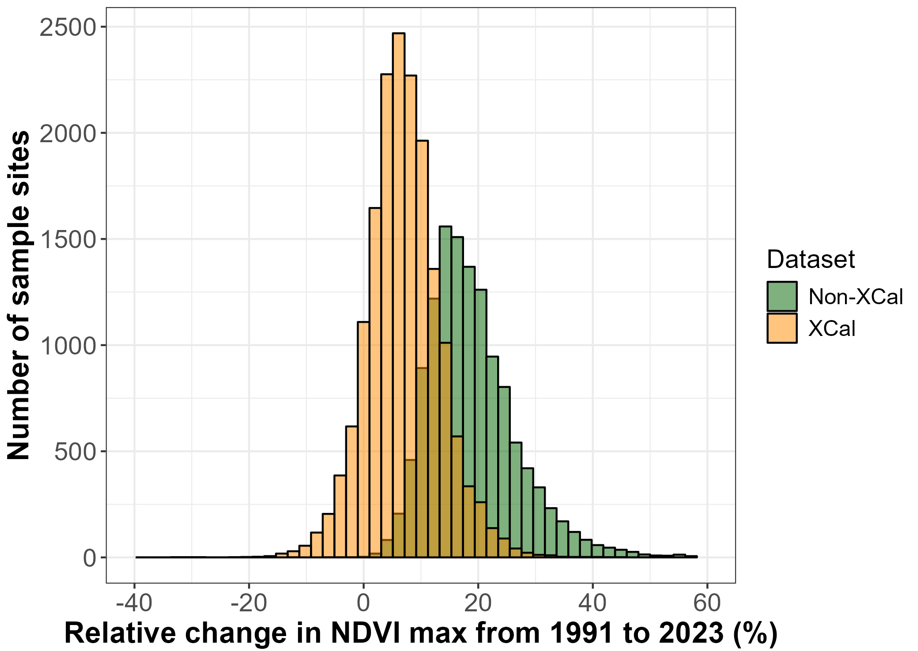
 Figure S4:** Histograms of relative NDVI_Max_ change per pixel between 1991 and 2023—the first and last years of data collection. The orange histogram shows NDVI values with cross-calibration applied; the green histogram shows values without cross-calibration.

**Landsat Phenological spline fitting**

From the cross-calibrated data we quantified the growing season characteristics using *LandsatTS*. This process involved iteratively fitting cubic splines to pixel measurements pooled over a seven-year moving window within the growing season. Further details can be found in the supplementary materials (Figure S5). Observations were exponentially weighted by distance in number of years from the focal year, so that observations from the focal year were most important in calculating its spline; observations outside the focal year (but inside the focal window) are thus used to bolster the number of datapoints through which to fit the spline. Outliers were excluded and the splines refitted until all observations were within a 30% bound of the fitted spline. If there were fewer than ten observations in the focal window, the spline was not fitted. As a further precaution, we removed pixels whose splines didn’t reach a peak before the date of the final observation.


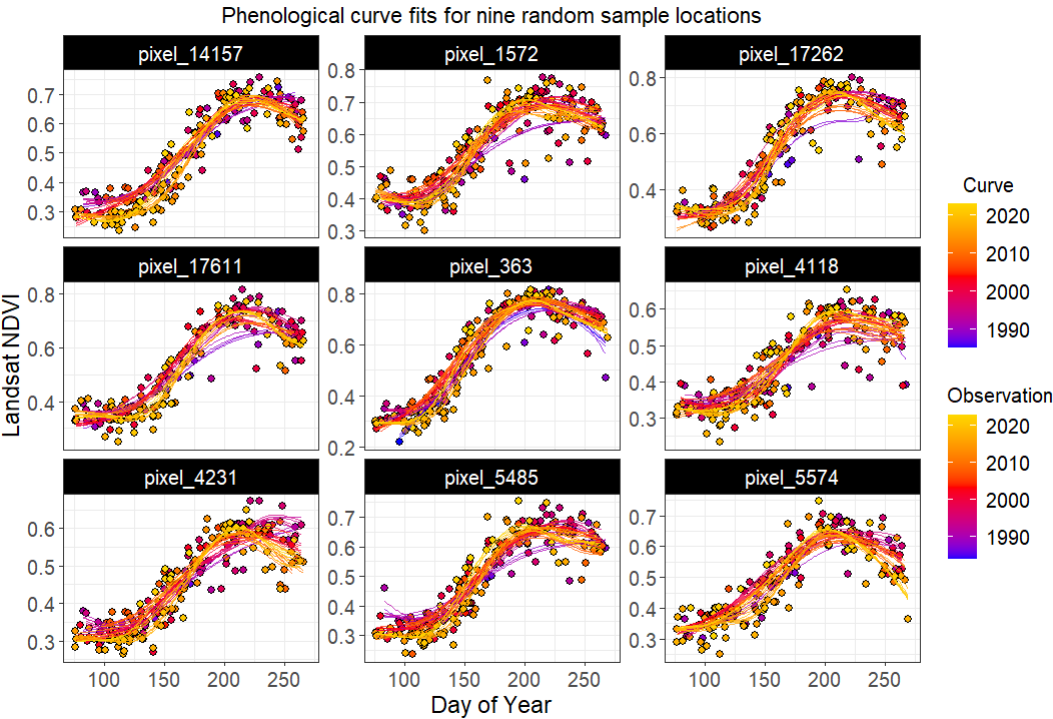
**Figure S5:** Seasonal NDVI progression for each year from 1985 to 2023 for nine randomly selected pixels in the Isle of Rum study area. Points and their corresponding fitted phenological curves (cubic splines) are colour coded by year. Plot produced using *LandsatTS* package prior to removal of pixels from splines which didn’t reach a peak.

As well as NDVI_Max,_ we also estimated NDVI_MaxDOY_, the day that the peak of the spline (i.e., NDVI_Max_) was reached. However, exploratory analysis led to concerns over the veracity of this metric for our dataset: a significant relationship was found between the number of cloud-free observations and the estimated NDVI_MaxDOY_ for each year (Figure S6). We therefore decided to exclude this metric from our analysis. No such relationship existed for the vegetation index NDVI_Max_.

A significant relationship was found between the number of cloud-free observations in a given year and its estimated NDVI_MaxDOY_. We therefore decided to exclude this metric from our analysis. No such relationship existed for NDVI_Max,_.

**
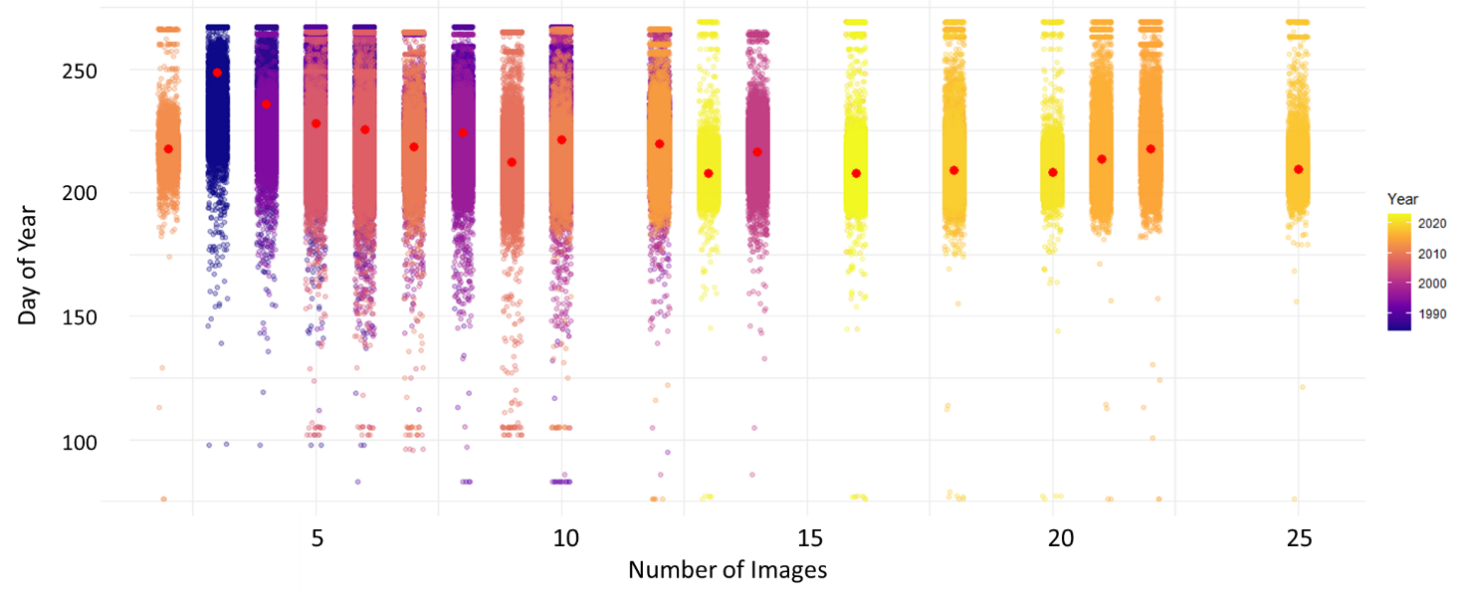
**

**Figure S6:** Annual NDVI_MaxDOY_ model predictions compared against the number of images available for each year. Colour depicts year; red dots overlaid indicate the estimated NDVI_MaxDOY_ for that year. A significant negative relationship was detected: the more images available, the lower the estimate of NDVI_MaxDOY_.

**MODIS metrics**

Annual ‘phenometrics’ are provided at six stages (Figure S7), calculated as the day of year on which a percentage threshold of the EVI_Amp_ was first or last crossed: Greenup (first crossed 15% of the EVI_Amp_), MidGreenup (50%), Maturity (90%), Senescence (last crossed 90% EVI_Amp_), MidGreendown (50%) and Dormancy (15%). We used MidGreenup and MidGreendown as the most reliable triggers of phenology (Friedl et al., 2022). The day at which EVI reached its peak was missing from the data, so we used Maturity (EVI_MaturityDOY_) as a proxy.


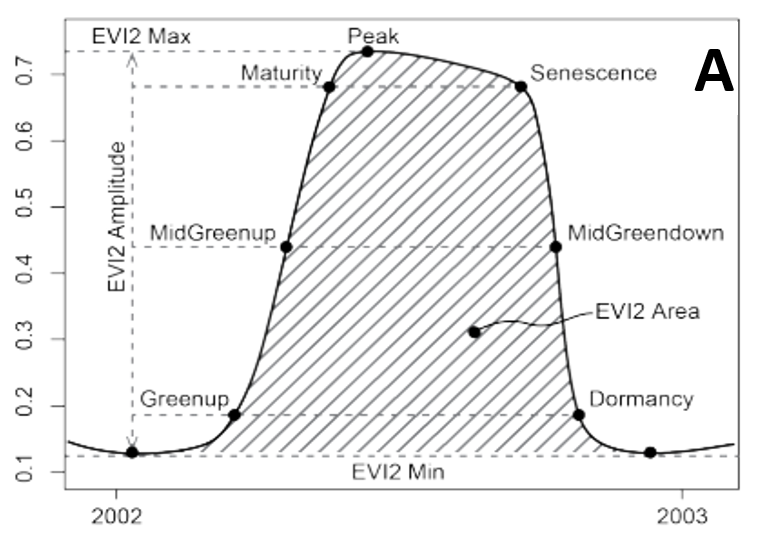


**Figure S7:** Diagram of the vegetation index and phenological metrics outputted by the MODIS MCD12Q2 product, taken from the product user guide (Friedl et al., 2022).

**Landsat Results**

Wet grassland exhibited a significantly stronger increase in NDVI_Max_ over time compared to acid grassland, while blanket bog and wet heath showed significant negative interactions, indicating a slower increase in NDVI_Max_ in these groups which are less preferred by the deer. Other interaction terms were not statistically significant. These results suggest that temporal trends in vegetation greenness are not uniform across vegetation groups, with wet grassland in particular greening more rapidly than other groups (Figure S8). Note this is the same figure as Figure 6 in the main text, but zoomed in and with the 95% confidence intervals removed to allow the reader to see the difference in slopes between vegetation groups.


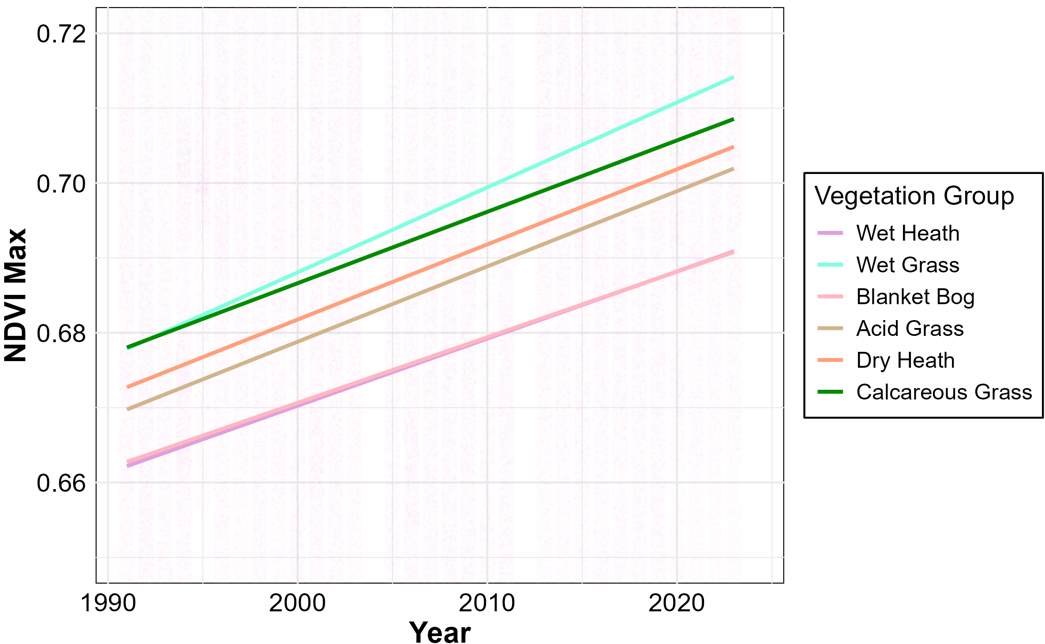
 **Figure S8:** Model estimates of NDVI_Max_ by vegetation group from Landsat data. Individual pixel estimates are coloured by vegetation group. Trendline predictions from the model with vegetation group and year interactions are overlaid.
